# Bibliometric Analysis of Whole-Body MRI from 2015 to 2025 Across Clinical Applications, Quantitative Imaging, and Artificial Intelligence

**DOI:** 10.3390/jimaging12080366

**Published:** 2026-08-08

**Authors:** Jonathan Lee, Christian Lee, Eric D. Cyphers, Casey Bonzell, Simon Kim, Michael Repajic, Reza Assadsangabi, Thomas G. Clifford, Vinay Duddalwar, Bryce D. Beutler

**Affiliations:** 1 Department of Radiology, Los Angeles General Medical Center, Los Angeles, CA 90033, USA; 2 Department of Radiology, Keck School of Medicine of USC, Los Angeles, CA 90033, USA; 3 Radiomics Lab, Department of Radiology, University of Southern California, Los Angeles, CA 90033, USA; 4 Alfred E. Mann Department of Biomedical Engineering, USC Viterbi School of Engineering, Los Angeles, CA 90089, USA; 5 Institute of Urology, University of Southern California, Los Angeles, CA 90033, USA

**Keywords:** whole-body MRI, diffusion-weighted imaging, bibliometrics, radiomics, artificial intelligence, oncologic imaging, musculoskeletal imaging, rare disease, pediatric imaging, quantitative biomarkers

## Abstract

Whole-body magnetic resonance imaging (WB-MRI) has become established for selected clinical indications and is increasingly studied across many fields including but not limited to oncologic, musculoskeletal, pediatric, and computational imaging applications. This study characterizes recent trends and the evolution of WB-MRI research using a bibliometric analysis reported using the PRISMA 2020 guidelines and identifies dominant clinical, quantitative, and artificial intelligence (AI) themes shaping the field. Publications were identified from Scopus, Web of Science, and PubMed using WB-MRI and its variant search terms and filtered to articles and reviews from 2015 through to 2025. Bibliometric analyses summarized publication output, contributors, citations, and keywords, and used exploratory keyword rules to classify major clinical and technical themes. Of 1511 included WB-MRI publications, annual output increased from 124 in 2015 to 180 in 2025. The journals with the highest publication counts were European Radiology, PLoS ONE, and the European Journal of Radiology. Parsed author affiliations most frequently represented the United States, Germany, and the United Kingdom. Research centered on oncologic applications, particularly myeloma and prostate cancer, alongside musculoskeletal disease, diffusion-weighted imaging, and AI-based segmentation. AI-related publications increased from 5 in 2015 to 20 in 2025. Broader clinical use of WB-MRI will require standardized acquisition, reproducible quantitative measures, and multicenter validation.

## 1. Introduction

Whole-body magnetic resonance imaging (WB-MRI) was originally positioned as a radiation-free method for surveying the body in a single examination, but its role has broadened substantially over the last decade. Contemporary WB-MRI now extends beyond descriptive anatomical screening, combining large-field-of-view imaging, diffusion-weighted imaging (DWI), fat–water separation, quantitative biomarkers, and structured response frameworks. In oncology, WB-MRI is increasingly discussed as a staging and response assessment platform, particularly when repeated surveillance is needed and radiation exposure is a concern [1,2]. In parallel, population-scale efforts such as the German National Cohort have demonstrated that whole-body MR protocols can be operationalized in research settings, supporting the feasibility of systematic whole-body imaging at scale [3].

The clinical appeal of WB-MRI is straightforward: it can depict and evaluate marrow, soft tissue, lymphatic, visceral, and musculoskeletal disease without ionizing radiation. This is especially relevant for pediatric patients, hereditary cancer syndromes, chronic inflammatory disease, and malignancies requiring repeated imaging. Recent cancer screening guidelines reflect this, framing WB-MRI as a potentially useful tool in selected high-risk groups rather than the general population [1,4]. The pediatric literature is more divided, demonstrating both the promise of broad anatomic coverage and the practical limitations of acquisition time, sedation, protocol design, and incidental findings [5]. As a result, WB-MRI sits at the intersection of diagnostic radiology, oncology, pediatrics, musculoskeletal imaging, and imaging informatics. This disease-focused rationale should be distinguished from elective MRI screening of asymptomatic general populations, where incidental findings and downstream testing remain important concerns.

WB-MRI is also technically heterogeneous. Some protocols emphasize anatomical T1-weighted and short tau inversion recovery imaging. Others rely heavily on DWI, particularly whole-body DWI (WB-DWI) with background body signal suppression. From a practical acquisition standpoint, contemporary oncologic WB-MRI protocols commonly cover the skull base to mid-thigh, analogous to PET/CT coverage, with vertex-to-feet imaging reserved for select indications. Additionally, modern protocols can combine DWI with morphologic T1- and T2-weighted imaging in approximately 30–45 min [1]. Quantitative WB-DWI requires specific guidance regarding acquisition, quality control, post-processing, and analysis, because apparent diffusion coefficient (ADC) measurements vary across scanners and reconstruction methods [6,7]. A UK quantitative WB-DWI workgroup illustrates the scale of this challenge, standardizing parameters that range from slice thickness, fat suppression technique, minimum and maximum b-values, head-to-mid-thigh coverage, setup optimization with a test object, and regular quality-control testing [6]. These technical issues matter clinically. Without reproducible acquisition and reporting, quantitative WB-MRI biomarkers cannot be reliably translated into multicenter trials or routine treatment response workflows. These technical complexities are precisely why a bibliometric map of the field is valuable: understanding which acquisition approaches and disease applications dominate the literature helps identify where consensus has formed and where methodological fragmentation persists.

A bibliometric analysis is useful in this setting because the field is broad, unevenly distributed, and rapidly changing. Traditional narrative reviews are well suited for summarizing specific disease indications, while scoping reviews and meta-analyses are better suited to structured evidence synthesis or pooled diagnostic performance within narrower clinical questions. These methods do not, by themselves, show how publication volume, journal distribution, international collaboration, keyword networks, and emerging methods have evolved across the entire WB-MRI literature. A reproducible bibliometric approach treats the literature as a dataset and maps dominant applications, journals, contributors, geographic links, and methodological anchors. Unlike disease-specific narrative or systematic reviews, and unlike prior PET/MR bibliometric work, this study compares these dimensions across the full WB-MRI corpus.

This study analyzed WB-MRI publications from 2015 through to 2025, with attention to clinical applications, quantitative imaging, and AI. Directed toward a radiology audience, this analysis emphasizes clinically interpretable findings: which applications are most mature, which methods are emerging, and where future studies are needed to move WB-MRI from a promising platform to a standardized diagnostic option.

## 2. Materials and Methods

### 2.1. Study Design

This study was designed as a bibliometric analysis of the WB-MRI literature published from 2015 through to 2025. Record identification, filtering, deduplication, and inclusion are reported in a PRISMA 2020 flow diagram generated in R 4.6.1 using the PRISMA2020 package version 1.1.4 [8,9]. The objective was to characterize publication volume, citation patterns, journal distribution, geographic representation, author productivity, and keyword structure. Articles and reviews were included; conference abstracts, editorials, corrections, and other ineligible document types were excluded when identifiable from database metadata. The study did not involve patient-level data, imaging data, or human subjects research.

### 2.2. Data Sources and Search Strategy

Records were collected from Scopus, Web of Science, and PubMed using search terms including “whole-body MRI”, “WB-MRI”, “WBMRI”, “whole-body magnetic resonance imaging”, “whole-body diffusion-weighted imaging”, “whole-body DWI”, and “diffusion-weighted whole-body imaging with background body signal suppression (DWIBS)”. Scopus and Web of Science records were used for richer bibliometric metadata, while PubMed improved biomedical coverage and supported PMID-based Zotero import. The search covered the complete calendar years 2015 through 2025 and was filtered to articles and reviews when document-type metadata were available. No separate language restriction was applied beyond database indexing and metadata availability. Citation counts reflect the May 2026 data export and were available primarily for Scopus-sourced records.

**Figure 1:**
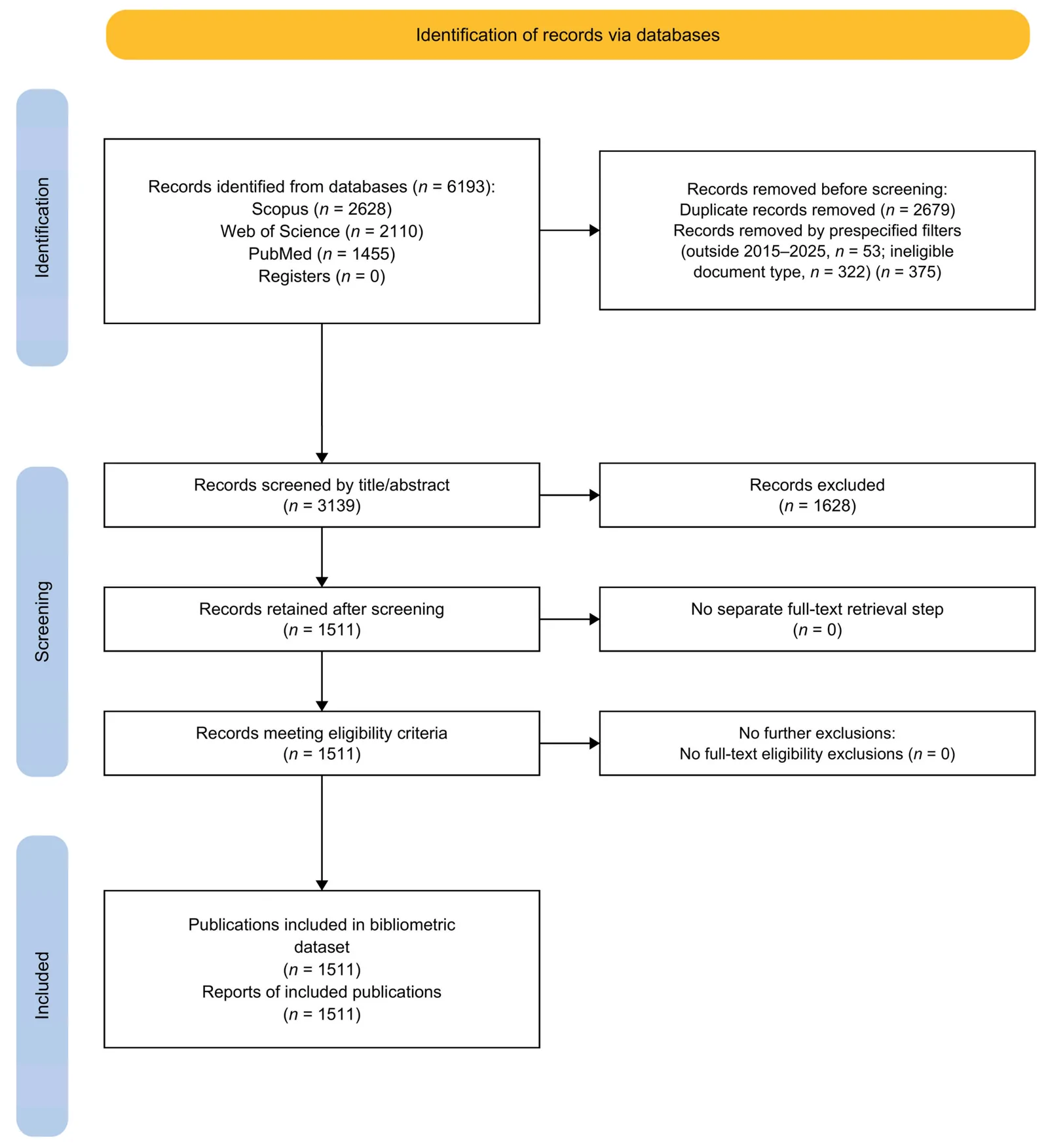
PRISMA 2020 flow diagram for database identification and bibliometric record selection. The diagram was generated with the PRISMA2020 R package. Of 6193 identified records (Scopus, *n* = 2628; Web of Science, *n* = 2110; PubMed, *n* = 1455), 2679 duplicates, 53 records outside 2015–2025, and 322 records with ineligible document types were removed. Title and abstract screening was then performed for 3139 unique records. 3074 had an abstract and 65 were assessed using title and bibliographic metadata only. Screening excluded 1628 records: 1556 without substantive WB-MRI use or evaluation, including incidental, scanner-only, and unavailable-only mentions; 60 ineligible publication types; and 12 WB-MRI acronym collisions. The final corpus included 1511 publications.

The search strategy was intentionally broad to maximize capture of clinically relevant literature, recognizing that WB-MRI terminology varies across disease domains. For example, myeloma studies often use the Myeloma Response Assessment and Diagnosis System (MY-RADS) and WB-MRI language, prostate cancer studies may use the Metastasis Reporting and Data System for Prostate Cancer (MET-RADS-P) or whole-body diffusion-weighted imaging (WB-DWI) terminology, and pediatric or hereditary syndrome studies may describe surveillance MRI without using the standard WB-MRI search terms. The search and screening workflow is summarized in Figure 1. Complete database-specific search strategies and additional methodological details are provided in the Supplementary Materials.

### 2.3. Eligibility Criteria and Record Screening

Eligible records were articles or reviews that addressed WB-MRI, WB-DWI, whole-body PET/MRI, whole-body quantitative MRI, or disease applications in which WB-MRI was a meaningful component of the study. Eligibility was determined from bibliographic metadata, including titles and abstracts; 3074 records had an abstract, and 65 were assessed using titles and bibliographic metadata only. For this bibliometric analysis, the final corpus reflects records captured by the predefined search strategy, publication-year limits, document-type filters, metadata standardization, and deduplication process. After metadata filtering and deduplication, 3139 records underwent title and abstract relevance screening. Records were retained when the title or abstract described direct use, evaluation, analysis, or repeated study-level discussion of WB-MRI or a prespecified variant. Scanner-only mentions, incidental or list-only mentions, and WB-MRI acronym collisions with weight-bearing MRI or water-bolus MRI were excluded. Full-text screening was not performed because the analysis classified bibliographic records rather than synthesizing clinical outcomes, and the study did not include risk-of-bias assessment or outcome-level evidence synthesis.

### 2.4. Dataset Selection, Database Comparison, and Deduplication

The three searches yielded 6193 records. Prespecified filters removed 53 records outside 2015–2025 and 322 ineligible document types; hierarchical deduplication removed 2679 duplicates, leaving 3139 unique records for relevance screening. Screening excluded 1628 records, and the final analytic corpus contained 1511 publications. The deduplication process was hierarchical: DOI was used first when available, followed by PMID, followed by a normalized title-year-first author key. This approach was necessary because the same paper often appeared across Scopus, Web of Science, and PubMed with slightly different metadata. Scopus served as the most complete source for citation counts and keyword fields. Database source counts, screening exclusions, and the final included corpus are displayed in Figure 1.

### 2.5. Data Extraction and Standardization

Extracted metadata included title, year, journal, authors, first author, DOI, PMID, document type, citation count, author keywords, index keywords, affiliation strings, and database source. Records were stored as a standardized tabular dataset for analysis. Zotero (Version 9.0.4) was used separately for citation management. Titles were normalized for deduplication, but exact titles were retained for citation verification. Author names were standardized for author productivity analysis by harmonizing capitalization, punctuation, whitespace, and author-initial formatting to reduce duplicate representations of the same author. Country fields were parsed from affiliation strings using rule-based matching against country names and aliases. Institution counts were derived from the first listed affiliation using heuristic text parsing for terms such as “University,” “Hospital,” “Institute,” “Centre,” “Center,” “Clinic,” and “Medical Center.” Registry identifiers such as Research Organization Registry (ROR) or Global Research Identifier Database (GRID) identifiers were not present in the exports, so institution-name variants could not be resolved reliably.

### 2.6. Bibliometric and Network Analyses

Annual publication counts, citation summaries, keyword frequencies, and the highest-publishing journals, countries, institutions and authors were calculated from the standardized dataset. Figure generation was performed in Python 3.11 using pandas 2.2.3 for tabulation, Matplotlib 3.10.0 for plotting, and NetworkX 3.4.2 for country collaboration analysis. Keyword frequencies were summarized in a ranked display of the 30 most frequent substantive keywords. Keyword co-occurrence and country collaboration files were also exported in VOSviewer-compatible tabular formats. In interpreting these results, special attention was given to the clinical meaning of clusters. For example, “multiple myeloma,” “bone marrow,” and “diffusion-weighted imaging” were interpreted as a mature oncologic and quantitative imaging cluster; “radiomics,” “segmentation,” and “deep learning” were interpreted as computational and AI-related clusters.

### 2.7. Theme Tagging and Subgroup Analyses

Exploratory theme tags identified publications related to oncology, musculoskeletal imaging, rare disease, pediatrics, DWI, quantitative biomarkers, radiomics, segmentation, and AI. Prespecified keyword rules were applied to titles, author keywords, and index keywords. No independent manual validation sample or inter-rater reliability statistic was obtained. The thematic results should therefore be interpreted as exploratory, reproducible rule-based classifications rather than adjudicated categories. For DWI and quantitative imaging, consensus and technical guidance papers were prioritized [6,7]. For AI and segmentation, frequently cited methodological and recent deep-learning papers were selected [10,11,12].

### 2.8. Reproducibility and Data Availability

Derived metadata, analysis scripts, aggregate tables, theme rules, and figure-generation code are available from the corresponding author upon reasonable request, subject to database licensing restrictions.

## 3. Results

### 3.1. Search Results, Database Coverage, and Included Literature

**Figure 2:**
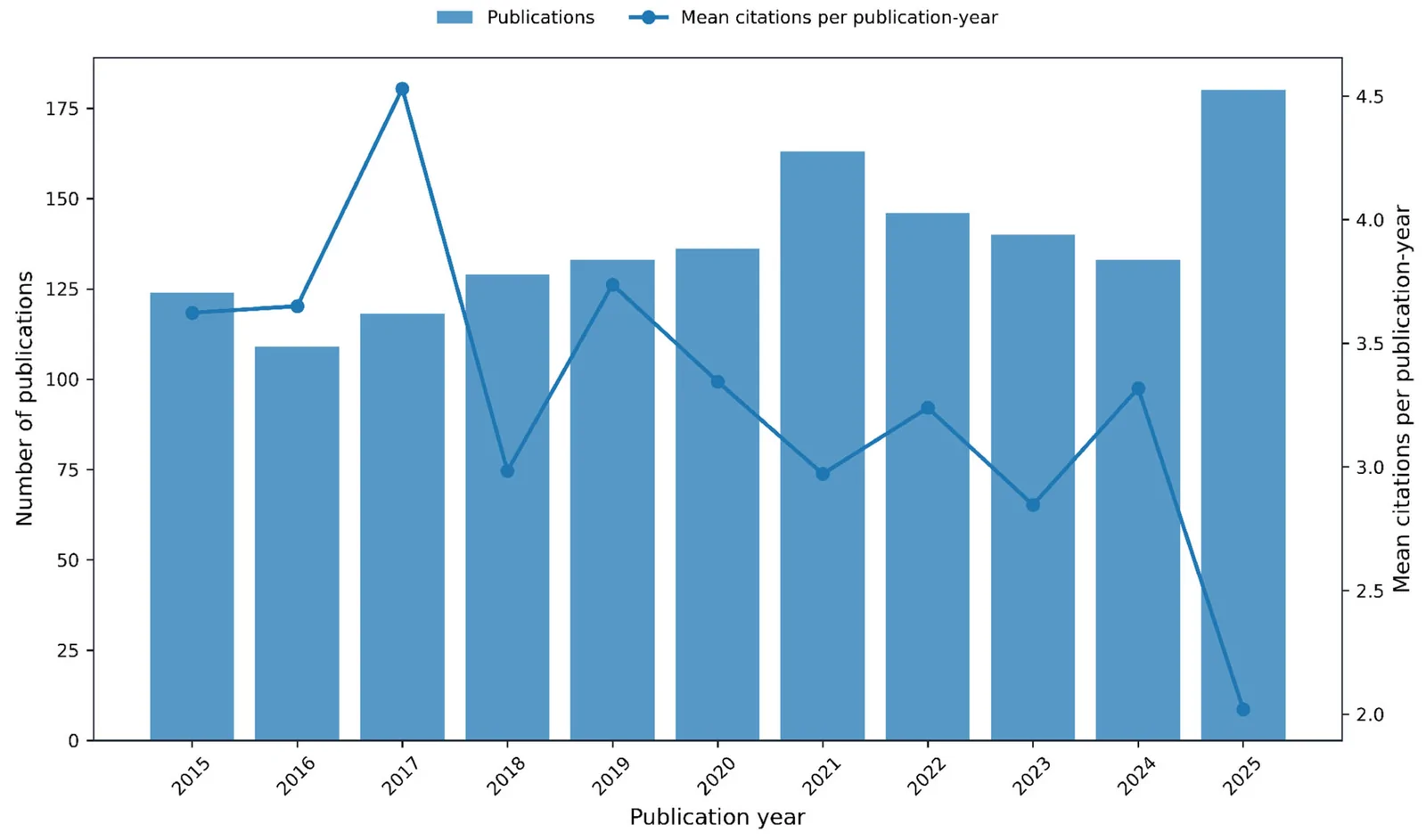
Annual WB-MRI publication volume and mean citations per publication-year (2015–2025). Bars show annual publication counts. The line shows the mean time-normalized citation rate among records with citation data.

The final bibliometric dataset included 1511 WB-MRI publications from 2015 through to 2025 after title and abstract relevance screening. Annual publication counts increased overall but were non-monotonic, rising from 124 in 2015 to 133 in 2024, with an interim high of 163 in 2021. A total of 180 publications were indexed in 2025. Annual publication and citation patterns are summarized in Table 1 and Figure 2. The dataset comprised 1271 articles (84.1%), 197 reviews (13.0%), 2 records labeled as both article and book chapter (0.1%), and 41 records without a recoverable document-type label (2.7%). Consensus statements and guidelines were not encoded as a separate document type and therefore could not be counted reliably. Publications appeared in major radiology, oncology, nuclear medicine, pediatric neuromuscular disease, and imaging informatics journals. These findings indicate that WB-MRI publications span multiple clinical and technical domains rather than a single disease area.

**Figure 3:**
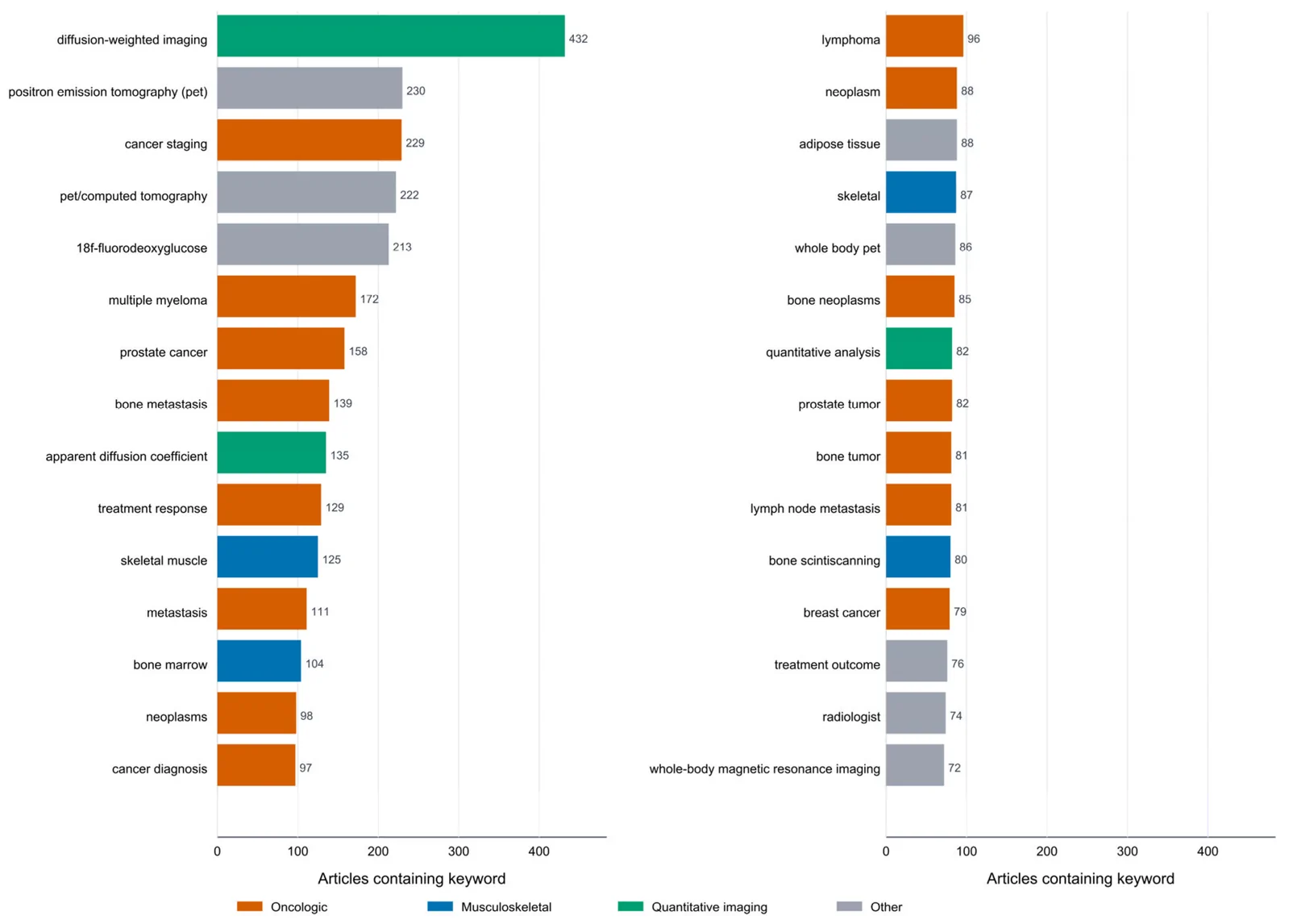
Thirty most frequent substantive keywords in the WB-MRI literature. Keywords are ranked in two panels after removal of generic demographic, publication-type, and database-indexing descriptors. Colors denote exploratory rule-based groups: oncologic, musculoskeletal, quantitative imaging, and other.

**Figure 4:**
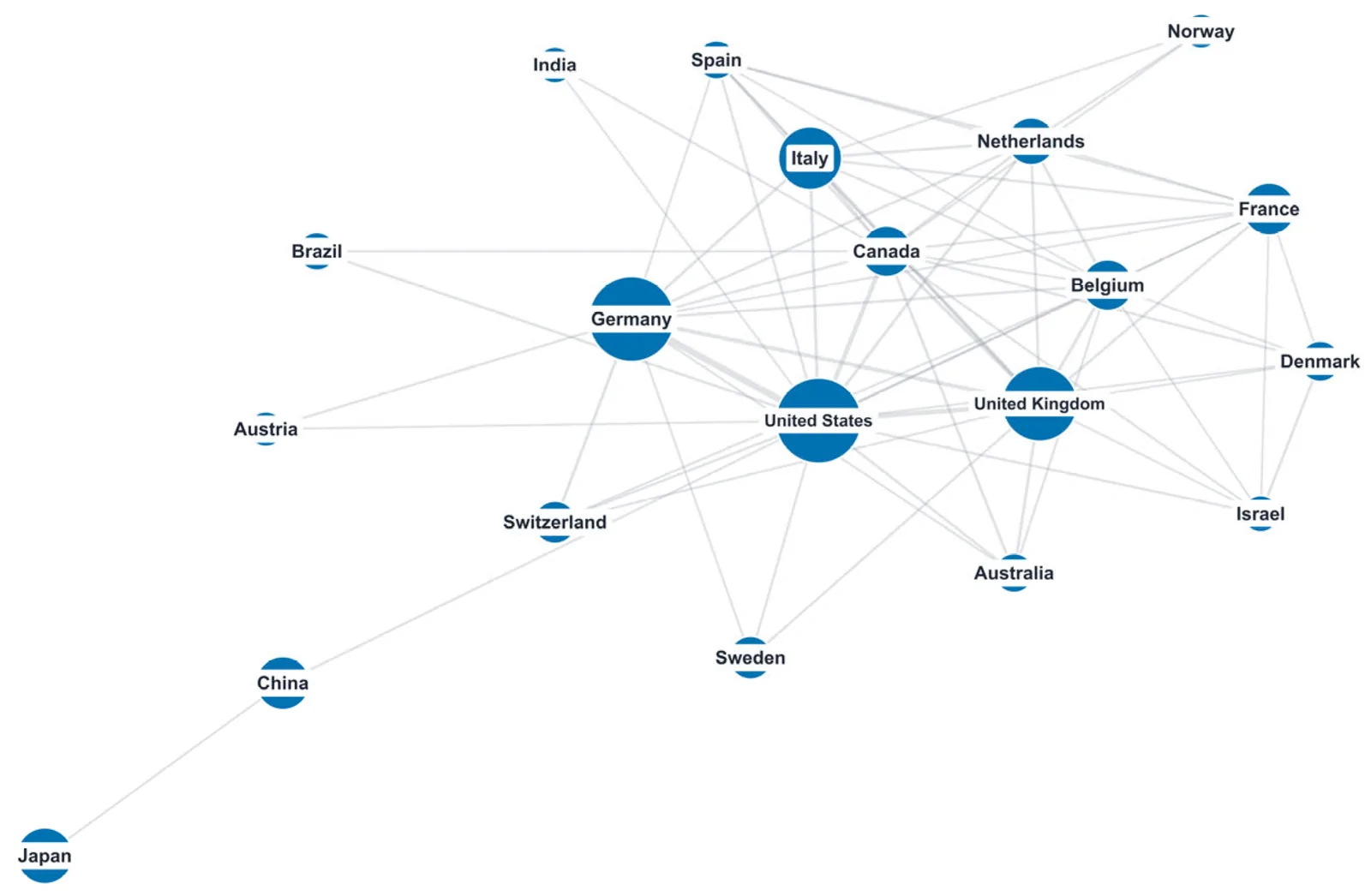
International collaboration network in whole-body MRI research. Nodes correspond to countries, with node size proportional to the number of publications originating from that country. Edges represent co-authorship links between countries. Thicker edges indicate stronger collaboration. Germany, the United States, and the United Kingdom occupy central positions, with numerous collaborations between European countries and North America. The static visualization summarizes aggregate collaboration structure across 2015–2025.

**Figure 5:**
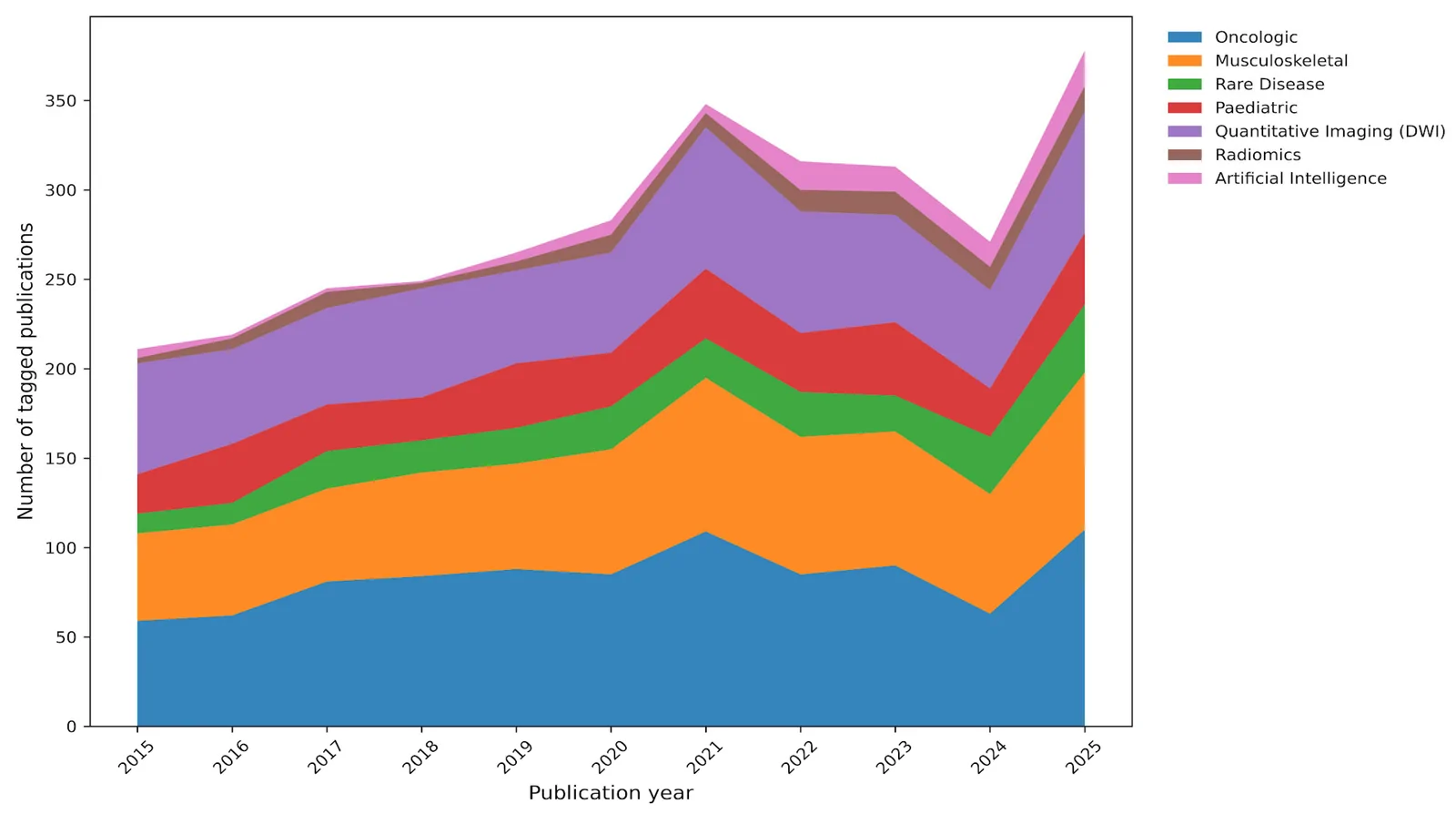
Exploratory thematic trends in WB-MRI publications. The stacked area chart shows overlapping annual keyword-rule tags for oncologic, musculoskeletal, rare disease, pediatric, quantitative imaging (DWI), radiomics, and artificial intelligence publications.

**Figure 6:**
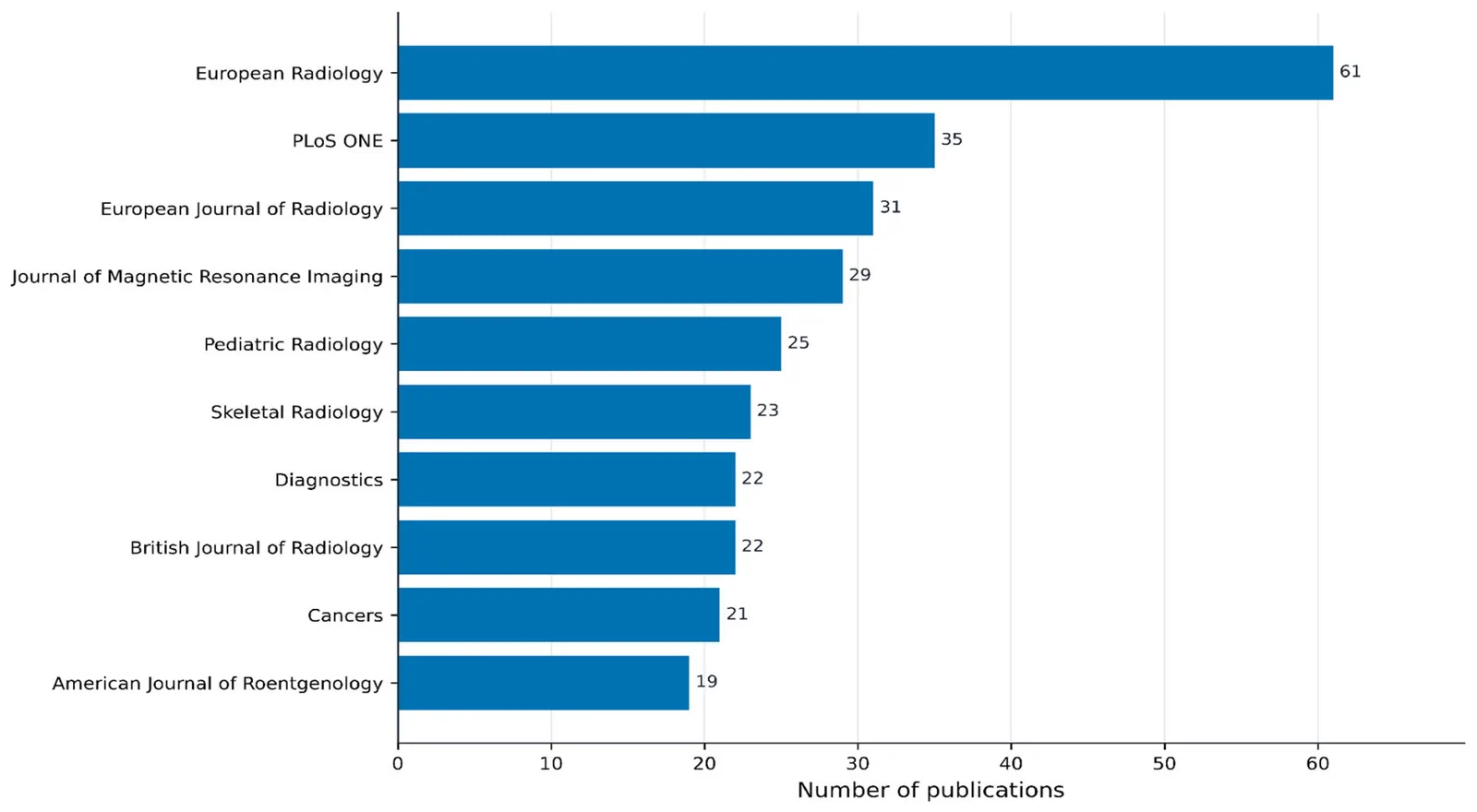
Top journals publishing WB-MRI research (2015–2025). Bars show the ten journals with the highest number of included publications, ordered by publication count. The leading journals were European Radiology, PLoS ONE, and European Journal of Radiology.

The journals with the highest publication counts were European Radiology (61 publications), PLoS ONE (35), European Journal of Radiology (31), Journal of Magnetic Resonance Imaging (29), Pediatric Radiology (25), and Skeletal Radiology (23). The full top-journal distribution is shown in Table 2. The principal graphical summaries of keyword frequency, international collaboration, thematic trends, and journal output are presented in Figure 3, Figure 4, Figure 5 and Figure 6, respectively. These results are consistent with the dual identity of WB-MRI as both a clinical radiology tool and a technical MRI research domain. Publications in Radiology, Radiographics, Blood, Journal of Clinical Oncology, The Lancet Oncology, and European Urology accounted for several influential clinical papers, particularly in multiple myeloma and prostate cancer [2,13,14].

### 3.2. Annual Publication Trends

Annual publication volume increased from 124 records in 2015 to 133 in 2024, with an interim high of 163 in 2021; 180 publications were indexed in 2025. Raw citation totals varied by publication age. After dividing each record’s citations by years since publication, mean citations per publication-year ranged from 2.85 to 4.53 during 2015–2024 and were 2.02 for 2025. The corresponding medians ranged from 1.67 to 3.11 and were 1.00 for 2025. Because citation coverage was incomplete outside Scopus and the 2025 accumulation interval was shorter, these values describe records with available counts rather than the entire corpus.

The temporal pattern also reflects changes in the field. Earlier publications were heavily concentrated in clinical staging, myeloma guidelines, WB-DWI, and population-based imaging feasibility, as reflected in Figure 5. Later publications more often carried AI, automated segmentation, response-assessment, cancer-predisposition, and rare-disease tags [12,15,16,17]. The AI increase plausibly reflects broader availability of deep-learning methods and large imaging datasets, although this bibliometric design cannot attribute causality.

### 3.3. Leading Countries, Institutions, and Authors

The United States, Germany, and the United Kingdom were the most frequent countries represented by parsed affiliations. Comparing mean annual affiliation counts in 2021–2025 with 2015–2020, China increased by 57.1%, Canada by 55.7%, and Japan by 46.4%, versus 37.1% for the United States, −5.1% for Germany, and 18.9% for the United Kingdom (Table 3). Figure 4 shows aggregate international co-authorship links. Because the network pools the full period, it cannot determine whether specific collaborations intensified over time. Population-level imaging studies from Germany contributed to the literature and support the feasibility of large-scale whole-body MR acquisition [3].

Author productivity was concentrated. Bamberg, Schlett, Koh, Tunariu, and Peters were among the most frequent authors, consistent with the prominence of population-based WB-MRI cohorts, oncologic WB-DWI groups, and disease-specific reporting frameworks. This pattern suggests that WB-MRI research has been driven by relatively specialized groups with expertise in protocol development, oncology applications, diffusion-weighted imaging, and multicenter trials. Concentrated expertise may create methodological continuity, but it also underscores the need to broaden participation across institutions, scanner vendors, and healthcare systems.

### 3.4. Citation Analysis

The most-cited publication in the screened dataset was the 2016 prospective observational study of surveillance in germline TP53 mutation carriers by Villani et al., with 448 citations in the May 2026 export [18]. Other highly cited publications included the MET-RADS-P recommendations and the MY-RADS consensus recommendations, reflecting the influence of standardized oncologic WB-MRI frameworks.

The most-cited clinical papers clustered around myeloma, cancer predisposition, and oncologic staging. The myeloma literature was particularly influential, as it included consensus guidance on MRI in myeloma management, the MY-RADS acquisition and reporting framework, and European Myeloma Network recommendations [2,13,19]. Hereditary cancer papers were also highly cited, especially Li–Fraumeni syndrome surveillance studies and guidelines [18,20,21,22]. These citation patterns indicate that WB-MRI has had the greatest clinical influence where repeated imaging, marrow assessment, and radiation reduction are especially important.

In an exploratory comparison of overlapping themes among records with citation data, mean (median) raw citations were 21.7 (11.0) for oncology, 20.0 (10.0) for musculoskeletal imaging, 20.0 (10.0) for quantitative imaging/DWI, and 19.2 (10.0) for AI. After normalization by years since publication, the corresponding means (medians) were 3.49 (2.33), 3.32 (2.29), 3.15 (2.00), and 4.78 (3.17) citations per publication-year. Thus, AI had the highest mean annualized rate but was represented by only 91 records with citation data. Overlapping tags and incomplete citation coverage preclude causal or quality inferences.

### 3.5. Keyword Co-Occurrence and Thematic Clusters

After removal of generic demographic, publication-type, and indexing terms, the ranked keywords emphasized diffusion-weighted imaging, apparent diffusion coefficient, multiple myeloma, bone metastasis, cancer staging, treatment response, and prostate cancer (Figure 3). Disease-specific terms were not included in the primary search string. Their prominence therefore reflects the retrieved WB-MRI corpus rather than deliberate disease enrichment. 

Several key thematic clusters were identified. A myeloma cluster was centered on bone marrow, focal lesions, DWI, response assessment, and diagnostic criteria [23,24,25]. A prostate cancer cluster included metastatic disease, MET-RADS-P, bone metastasis volume, and advanced imaging pathways [14,26,27]. A musculoskeletal cluster included spondyloarthritis, enthesitis, non-bacterial osteitis, myopathy, and muscle MRI [28,29,30]. The exploratory AI group included classification or detection, segmentation or localization, reconstruction or correction, and image synthesis [12,31,32]. No AI-tagged publication matched the prespecified natural-language-processing terms (“natural language,” “NLP,” or “language model”).

### 3.6. Clinical Applications of Whole-Body MRI

Oncology remains the dominant clinical application of WB-MRI, as reflected in the exploratory trends in Figure 5. Currently, multiple myeloma is the most mature use case, as disease burden is frequently marrow-based, response assessment can be lesion-based, and repeated imaging is common. Earlier consensus guidance established the role of MRI in myeloma management [13], and MY-RADS has since standardized acquisition, interpretation, and response assessment for WB-MRI in myeloma [2]. MY-RADS also provides practical reading guidance: high b-value DWI maximum-intensity-projection images can help display global tumor burden and regional disease distribution; however, they should not be interpreted alone because T2 shine-through, motion-related signal loss, sparse disease patterns, and benign marrow or osseous processes can mimic or obscure disease [2]. Later publications have extended this framework into diagnostic criteria, treatment monitoring, quantitative biomarkers, and response assessment [23,24,25].

Prostate cancer represents another important oncologic application. MET-RADS-P provided practical guidance for acquisition, interpretation, and reporting in advanced prostate cancer [14,26]. Systematic review evidence also supports MRI for detecting bone metastases in prostate cancer [27].

More broadly, prospective imaging trials have compared WB-MRI with standard staging pathways in other oncologic diseases. In STREAMLINE C, WB-MRI produced similar metastatic staging accuracy to standard pathways in newly diagnosed colorectal cancer while reducing median staging time from 13 to 8 days, decreasing the median number of tests from two to one, and lowering mean per-patient staging costs from £285 to £216 [33]. In STREAMLINE L, WB-MRI similarly shortened staging in potentially radically treatable non-small-cell lung cancer by reducing median staging time from 19 to 13 days, lowering mean per-patient staging costs from £620 to £317, and achieving similar agreement with final multidisciplinary treatment decisions compared with standard staging pathways [34].

Comparative oncologic imaging studies have evaluated WB-MRI against PET/CT, CT, bone scintigraphy, and other whole-body modalities. The SKELETA trial compared planar bone scintigraphy, SPECT, SPECT/CT, 18F-NaF PET/CT, and whole-body 1.5T MRI with DWI for detection of bone metastases in high-risk breast and prostate cancer patients [35]. In lymphoma, WB-MRI with DWI was compared with PET/CT for staging newly diagnosed fluorodeoxyglucose (FDG)-avid lymphoma [36]. In suspected ovarian cancer, whole-body diffusion-weighted MRI was compared with CT for preoperative assessment [37]. These comparative studies show that WB-MRI is most useful when clinical questions require both broad anatomic coverage and high soft-tissue or marrow contrast.

Cancer-predisposition surveillance is an important application, whereas the pediatric tag remained smaller and fluctuated without a sustained monotonic rise (22 records in 2015, a high of 41 in 2023, and 40 in 2025). Li–Fraumeni syndrome is the most visible example, with prospective surveillance data, screening recommendations, meta-analysis evidence, and updated guidance supporting the role of WB-MRI in surveillance strategies [16,18,20,21,22]. In the baseline Li–Fraumeni surveillance meta-analysis, Ballinger et al. pooled 578 TP53 carriers from 13 cohorts in 6 countries and reported an estimated 7% detection rate for new localized primary cancers at baseline WB-MRI [21]. Surveillance recommendations for Von Hippel-Lindau and hereditary pheochromocytoma/paraganglioma syndromes further illustrate how WB-MRI can contribute to radiation-sparing surveillance in inherited cancer syndromes [38,39]. In neurofibromatosis type 1, pediatric tumor surveillance recommendations and newer multiparametric WB-MRI descriptions demonstrate the expanding role of MRI in syndromic tumor burden assessment [40,41].

Musculoskeletal and inflammatory applications are smaller in volume but remain a distinct and recurring theme in the corpus. A foundational *Radiology* review outlined musculoskeletal applications of WB-MRI [42]. In inflammatory arthritis, head-to-toe WB-MRI has been used to assess global inflammation and damage in psoriatic arthritis and axial spondyloarthritis [28], including assessment of enthesitis across peripheral and axial sites [29]. For chronic non-bacterial osteitis and chronic recurrent multifocal osteomyelitis, WB-MRI can detect multifocal lesions that may be clinically occult [17,43,44]. Neuromuscular disease applications include WB-MRI in facioscapulohumeral muscular dystrophy and standardized MYO-MRI protocols for genetic myopathies [30,45]. This subset of the literature demonstrates how WB-MRI has been used to map the extent of multifocal and systemic disease in a single diagnostic study.

### 3.7. Quantitative Imaging and Diffusion-Weighted MRI Trends

DWI is the principal quantitative method in WB-MRI, and its exploratory publication trend is shown in Figure 5. The UK quantitative WB-DWI technical workgroup recommendations and consensus guidance on DWI outside the brain provide the technical basis for using diffusion metrics in body imaging and oncology [6,7]. One challenge is that WB-DWI is not a single measurement; it is a chain of acquisition, reconstruction, segmentation, and analysis decisions. ADC estimates may differ across b-value schemes, body regions, scanner hardware, and post-processing pipelines. Recent work comparing ADC measurement strategies in WB-MRI for multiple myeloma illustrates that even seemingly small protocol choices can affect quantitative interpretation [46]. In a 55-patient study of multiple myeloma, ADC maps reconstructed from three b-values (50, 500, and 1000 s/mm^2^) produced slightly higher ADC values than two b-values (50 and 1000 s/mm^2^) for both bone lesions and healthy marrow, and 10 of 305 bone lesions crossed the MY-RADS malignancy threshold only on the three-b-value reconstruction [46].

Proposed quantitative WB-MRI biomarkers have been studied most extensively in myeloma and metastatic prostate cancer. In newly diagnosed symptomatic myeloma, quantitative WB-MRI biomarkers were associated with treatment response after bortezomib induction [24]. In this prospective cohort, focal-lesion signal fat fraction and ADC increased in treatment responders, while focal-lesion signal fat fraction was the strongest discriminator of response, with a reported AUC of 1.0 [24]. In prostate cancer, WB-DWI-derived bone metastasis volume was associated with overall survival, supporting the concept that tumor burden metrics may have prognostic value [26]. In this study, 43 eligible men with metastatic castration-resistant prostate cancer had a median total diffusion volume of 503.1 mL, and higher total diffusion volume was associated with poorer overall survival, with a hazard ratio of 1.74 (95% CI, 1.02–2.96; *p* = 0.035) [26]. Normal reference work, such as studies of ADC values in abdominal organs and bone marrow from WB-DWI, is also important because biomarker interpretation requires knowledge of expected physiologic variation by age, sex, and tissue type [47].

Body composition and muscle imaging represent another quantitative branch of WB-MRI. Studies comparing single-slice measures with whole-body skeletal muscle and adipose tissue volumes highlight both the efficiency and the limitations of simplified body composition metrics [48]. Automated segmentation of regional muscle volume using whole-body water–fat MRI demonstrates how quantitative whole-body datasets can support reproducible muscle and fat measurement [49]. For example, Karlsson et al. used whole-body water–fat MRI with multi-atlas segmentation of 10 manually segmented muscle groups and reported high agreement with manual segmentation, including whole-body intraclass correlation coefficients of 0.99 at 1.5 T and 0.97 at 3.0 T [49]. This work is potentially relevant for sarcopenia, obesity phenotyping, neuromuscular disease, and frailty imaging.

### 3.8. Artificial Intelligence and Computational Imaging Trends

Exploratory AI-tagged WB-MRI publications increased from 5 in 2015 to 20 in 2025; 92 publications received an AI tag across the full study period (Figure 5). The AI-tagged group was smaller than the oncologic, musculoskeletal, and quantitative-DWI groups. This topic is highly relevant, as AI models in WB-MRI often depend on segmentation of marrow, skeleton, lesions, muscle groups, and organs, and without appropriate evaluation metrics, automated methods can appear accurate while failing on clinically important lesion boundaries or small structures.

Recent AI studies have moved from general segmentation concepts toward WB-MRI-specific tasks. A 2025 multicenter study applied nnU-Net, an automated model for robust bone marrow segmentation, to 210 WB-MRI examinations from 207 patients across eight centers [12]. The model achieved mean Dice scores of 0.89 ± 0.13 and 0.88 ± 0.11 on two external test sets, with individual bone marrow space Dice scores ranging from 0.77 to 0.96 and 0.81 to 0.95, respectively [12]. A multicenter feasibility study evaluated automated detection of focal bone marrow lesions in patients with monoclonal plasma cell disorders [15]. The model was trained on 334 patients with 494 focal lesions and tested on internal and external multicenter cohorts, but performance decreased from internal to external testing, with mAP falling from 0.44 to 0.34 and sensitivity from 0.49 to 0.34 [15]. Deep-learning body region segmentation has been used to automate field-of-view prescription for WB-MRI [32] and a weakly supervised model was used to localize and delineate the skeleton, internal organs, and spinal canal on WB-DWI [11]. In hybrid imaging, a deep neural network was used to generate PET attenuation maps for whole-body time-of-flight FDG PET/MRI [31]. Exploratory subtopic rules classified 68 of 92 AI-tagged publications as classification/detection, 32 as segmentation/localization, 10 as reconstruction/correction, and 3 as image synthesis/generation. The image-synthesis group included MR-to-CT methods using multi-contrast inputs and a whole-body uncertainty-aware network [50,51].

These studies show that AI in WB-MRI is becoming more task-specific, but several barriers remain. Models need external validation, scanner diversity, transparent reporting, clinically meaningful error analysis, and integration into reporting workflows. The field should avoid assuming that high segmentation metrics automatically translate into better clinical decisions. Instead, future studies should connect AI outputs with reader time, lesion detection accuracy, response assessment consistency, clinical outcomes, and cost-effectiveness.

## 4. Discussion

### 4.1. Principal Findings

This bibliometric analysis found that, overall, WB-MRI research expanded substantially between 2015 and 2025. The final screened dataset included 1511 publications. Annual output increased from 124 records in 2015 to 133 in 2024, with an interim high of 163 in 2021; 180 publications were indexed in 2025. The literature is concentrated in radiology and MRI journals, but extends into oncology, hematology, rheumatology, pediatrics, neuromuscular disease, nuclear medicine, and imaging informatics. The highest-cited records included consensus guidelines and disease-surveillance recommendations.

These findings are consistent with broader trends in MRI-based whole-body and hybrid imaging research. A recent bibliometric analysis of PET/MR similarly demonstrated growth in publication volume, prominent contributions from the United States and Germany, and a shift from technical imaging topics toward clinical applications [52]. The PET/MR study mapped a hybrid modality, whereas disease-specific WB-MRI narrative and systematic reviews synthesize indications or diagnostic performance within narrower questions [1,5,53]. The present analysis contributes a cross-domain map of the full WB-MRI corpus, integrating temporal output, normalized citations, journals, geography, collaboration, and exploratory method themes. Its principal comparative finding is that oncologic applications and quantitative DWI remain the mature core, while AI-enabled workflow research is smaller but increasing. Oncology remains the central driver of WB-MRI research, with myeloma, prostate cancer, lymphoma, colorectal cancer, lung cancer, ovarian cancer, and hereditary cancer syndromes accounting for many of the most clinically influential papers [14,25,33,34]. Quantitative imaging and DWI are central to many WB-MRI publications, providing a framework for response assessment, tumor burden measurement, and proposed biomarker development [6,7,24]. AI has also become a more visible theme, with recent papers focusing on marrow segmentation, focal lesion detection, field-of-view prescription, and WB-DWI organ localization, all of which address practical bottlenecks in whole-body imaging workflows [11,12,15,32].

### 4.2. Whole-Body MRI as an Evolving Diagnostic Imaging Platform

WB-MRI is best understood as an evolving imaging platform rather than a single protocol. In oncology, it stages disease, measures marrow tumor burden, and monitors response. In musculoskeletal disease, it evaluates the distribution of inflammation, bone lesions, and muscle involvement. In pediatrics and inherited cancer syndromes, it provides repeated surveillance without ionizing radiation. In body composition research, it quantifies muscle and adipose tissue at scale. These applications share the same core advantage: broad anatomic coverage with high soft-tissue contrast.

The platform concept also explains why standardization is difficult. A myeloma protocol optimized for marrow focal lesions is not identical to a pediatric cancer predisposition surveillance protocol, a spondyloarthritis protocol, or a muscle fat quantification protocol. However, the literature suggests that disease-specific frameworks can improve consistency. Two examples of this are MY-RADS in myeloma [2] and MET-RADS-P in advanced prostate cancer [14]. Similar disease-specific reporting structures may be needed for pediatric surveillance, chronic non-bacterial osteitis, and neuromuscular disease.

### 4.3. Expansion in Oncologic, Musculoskeletal, and Rare Disease Applications

The strongest evidence base is in oncology. Myeloma has the clearest reporting framework and multiple influential guidance papers [13,19,23]. Prostate cancer has strong disease-specific reporting through MET-RADS-P and quantitative tumor burden work [14,26]. Prospective STREAMLINE trials in colorectal cancer and non-small-cell lung cancer provide important evidence that WB-MRI can be compared directly with standard staging pathways in newly diagnosed malignancy [33,34].

Multiple myeloma likely achieved guideline-level WB-MRI evidence earlier than many other applications because the disease is intrinsically marrow-based and frequently requires repeated response assessment. WB-MRI is therefore well matched to the biology and clinical workflow of myeloma, particularly when marrow lesion burden and treatment response are central questions. Other disease applications face greater barriers, including less uniform acquisition protocols, more heterogeneous disease patterns, and fewer multicenter studies linking WB-MRI findings to clinical decision-making or patient outcomes.

Cancer predisposition surveillance is another area where WB-MRI has a clinically intuitive role because the harms of repeated radiation exposure are particularly relevant. Li–Fraumeni syndrome has the deepest literature, including prospective surveillance, screening recommendations, meta-analysis, and updated guidance [16,18,20,21,22]. Hereditary pheochromocytoma/paraganglioma syndromes, Von Hippel–Lindau disease, and neurofibromatosis type 1 further demonstrate that WB-MRI can support surveillance in syndromes with multifocal or systemic tumor risk [38,39,40,41].

Musculoskeletal and inflammatory applications are less dominant numerically but remain a recurring theme for clinical radiology. WB-MRI can depict multifocal inflammatory lesions, enthesitis, osteitis, and muscle involvement. The literature in psoriatic arthritis and axial spondyloarthritis illustrates the potential of whole-body imaging to measure disease distribution beyond what is feasible with regional MRI alone [28,29]. Chronic non-bacterial osteitis is another strong use case because lesions may be multifocal and clinically silent [17,43,44]. Neuromuscular disease imaging demonstrates that WB-MRI can also serve as a pattern recognition and quantitative muscle assessment tool [30,45,53].

### 4.4. Growth of Quantitative Imaging and Proposed Biomarkers

Quantitative imaging is the bridge between visual interpretation and measurable disease burden. In WB-MRI, DWI and ADC are the most visible quantitative methods, while water–fat MRI and muscle volumetry are important for body composition and neuromuscular disease. The UK quantitative WB-DWI recommendations and broader DWI consensus work are important because they make clear that quantitative imaging requires protocol discipline [6,7]. Without standardization, ADC values and lesion volumes are difficult to compare across sites and trials.

The selected literature shows several directions for biomarker development. Myeloma papers have linked quantitative WB-MRI measures with treatment response [24,54]. Prostate cancer studies have evaluated WB-DWI tumor volume as a prognostic marker [26]. Body composition papers have used MRI to quantify skeletal muscle, adipose tissue, and regional muscle volumes [48,49]. However, clinical translation requires more than measurement reproducibility. Future studies must define thresholds, demonstrate outcome associations, and show whether quantitative metrics alter management.

### 4.5. Emergence of Artificial Intelligence, Radiomics, and Automation

AI is emerging at a time when WB-MRI may benefit from automation. Whole-body examinations generate large datasets, and manual segmentation of marrow, lesions, organs, or muscle groups is time-consuming. The literature now includes models for bone marrow segmentation, focal lesion detection, field-of-view prescription, and WB-DWI localization [11,12,15,32]. These are not abstract AI tasks. They address real bottlenecks in WB-MRI acquisition, interpretation, and quantitative analysis. Radiomics-based approaches have appeared consistently throughout the study period as a bridge between traditional quantitative imaging and deep-learning methods. However, radiomics publications remained less frequent than several other themes, likely reflecting the difficulty of standardizing whole-body radiomics workflows across protocols and scanners.

The methodological challenge is validation. A model that performs well in one center or scanner environment may fail when acquisition parameters, coils, patient body habitus, disease pattern, or reconstruction methods change, as demonstrated by the multicenter lesion detection study, in which performance decreased notably when switched from internal to external settings [15]. Segmentation metrics are necessary for evaluation, but they must be interpreted with clinical context [10]. In the near term, the most useful AI tools may be those that reduce repetitive tasks, improve consistency, or identify cases needing closer review rather than fully replacing radiologist interpretation.

### 4.6. Research Gaps and Future Priorities

The major gaps are not only technical, but are also translational. First, acquisition protocols remain heterogeneous across diseases, scanners, and institutions. Second, many papers evaluate diagnostic performance or technical feasibility, while fewer link WB-MRI findings to patient outcomes, clinical decisions, cost, or treatment changes. Third, quantitative and AI methods often lack external validation. Fourth, pediatric and hereditary surveillance raise unresolved questions about incidental findings, anxiety, follow-up burden, and cost.

Future studies should therefore be designed around clinical endpoints and implementation. For myeloma, this means testing whether MY-RADS-based response assessment improves treatment decisions or trial stratification [2,25]. The prospective iTIMM trial provides an example of this direction: among 70 myeloma patients undergoing WB-MRI before induction and at day 100 after autologous stem cell transplantation, residual disease on MY-RADS response assessment was associated with shorter progression-free survival, with median progression-free survival of 24 months versus 42 months and a hazard ratio of 2.09 [25]. For prostate cancer, it means validating WB-DWI tumor burden metrics across multicenter cohorts and integrating them with prostate-specific membrane antigen (PSMA) PET and systemic therapy pathways [14,26]. For pediatric and hereditary syndromes, it means balancing early detection with false positives, sedation risk, and surveillance burden [5,16]. For AI, it means moving from proof-of-concept segmentation to prospective workflow and reader studies [12,15].

### 4.7. Limitations

This study has several limitations. Scopus, Web of Science, and PubMed do not provide complete coverage of EMBASE, conference proceedings, grey literature, or non-English and regionally indexed publications. European subspecialty literature may therefore be unevenly represented. Citation counts were available primarily from Scopus and remain age-dependent despite the added citations-per-publication-year comparison. Relevance screening used a reproducible, conservative title-and-abstract rule set rather than independent dual-reviewer adjudication. 65 records lacked an abstract and were assessed from titles and bibliographic metadata only, so residual misclassification remains possible. Full-text screening was not performed because the study classified bibliographic records rather than synthesizing clinical outcomes. Document-type metadata separated articles, reviews, and unavailable types but did not reliably distinguish consensus statements or guidelines. The narrative discussion uses selected included papers for context and is not a systematic review of diagnostic accuracy or clinical effectiveness. Country and institution analyses relied on affiliation strings. ROR/GRID identifiers were unavailable, first-affiliation parsing can merge or split organizations, and the aggregate collaboration network does not measure temporal change in individual ties. Manufacturer was not a standardized bibliographic field, so vendor-level analysis could not be performed without full-text or imaging-protocol review. Finally, overlapping theme and AI-subtopic tags were assigned by keyword rules without an independent validation sample or inter-rater reliability testing and should be interpreted as exploratory.

## 5. Conclusions

WB-MRI research increased overall from 2015 through to 2025, although annual output was non-monotonic. Oncology, DWI, and quantitative imaging formed the core of the field, with multiple myeloma and prostate cancer showing the most mature disease-specific frameworks. Cancer-predisposition surveillance, musculoskeletal inflammation, non-bacterial osteitis, and neuromuscular imaging remained important, whereas pediatric output was smaller and variable rather than consistently increasing. Exploratory AI tags increased, led by classification/detection and segmentation/localization. Reconstruction and image-synthesis applications were less common.

The next stage of WB-MRI research should focus on harmonized acquisition, reproducible quantitative biomarkers, multicenter validation, and clinically useful AI. The literature now contains enough disease-specific and technical work to support more rigorous implementation studies. The goal should not be more WB-MRI publications alone, but better evidence showing when WB-MRI changes management, improves outcomes, reduces radiation burden, and provides value beyond standard imaging pathways.

## Figures and Tables

**Table 1:** Annual publication counts and time-normalized citation summary for the WB-MRI literature (2015–2025). Citation data were available primarily for Scopus-sourced records and reflect the May 2026 export. Citations per publication-year divide each record’s citation count by years since publication (minimum denominator, 1 year).

Year	Publications	Cumulative Percent	Median Citations per Publication-Year	Mean Citations per Publication-Year	Records with Citation Data
2015	124	8.2%	2.18	3.62	116
2016	109	15.4%	2.50	3.65	100
2017	118	23.2%	3.11	4.53	109
2018	129	31.8%	2.50	2.98	123
2019	133	40.6%	2.29	3.74	123
2020	136	49.6%	2.58	3.35	128
2021	163	60.4%	1.80	2.97	155
2022	146	70.0%	2.25	3.24	139
2023	140	79.3%	1.67	2.85	130
2024	133	88.1%	2.00	3.32	121
2025	180	100.0%	1.00	2.02	155

**Table 2:** Top ten journals publishing whole-body MRI research (2015–2025). For each journal, the number of included WB-MRI publications, percentage of total publications, and cumulative percentage are listed.

Rank	Journal	Publications	Percent of Total Publications	Cumulative Percent
1	European Radiology	61	4.04	4.04
2	PLoS ONE	35	2.32	6.36
3	European Journal of Radiology	31	2.05	8.41
4	Journal of Magnetic Resonance Imaging	29	1.92	10.33
5	Pediatric Radiology	25	1.65	11.98
6	Skeletal Radiology	23	1.52	13.50
7	British Journal of Radiology	22	1.46	14.96
8	Diagnostics	22	1.46	16.42
9	Cancers	21	1.39	17.81
10	American Journal of Roentgenology	19	1.26	19.07

**Table 3:** Top ten countries contributing to the WB-MRI literature and annualized growth. Countries are ranked by parsed affiliation count. Growth compares the mean annual affiliation count in 2021–2025 with 2015–2020. Counts are nonexclusive because multicountry publications contribute to each represented country.

Rank	Country	Country Affiliation Count	Growth in Mean Annual Affiliation Count, %
1	United States	300	+37.1%
2	Germany	299	−5.1%
3	United Kingdom	225	+18.9%
4	Italy	149	+46.9%
5	Japan	111	+46.4%
6	China	97	+57.1%
7	France	92	−11.7%
8	Belgium	86	+14.5%
9	Canada	85	+55.7%
10	Netherlands	69	−2.1%

## Data Availability

The original contributions presented in this study are included in the article/Supplementary Material. Further inquiries can be directed to the corresponding author.

## Supplementary Materials

The following supporting information can be downloaded at: https://www.mdpi.com/article/10.3390/jimaging12080366/s1. Detailed search strategies and additional methodological details are provided in the Supplementary Methods.
