# Supplementary material for: Bibliometric Analysis of Whole-Body MRI from 2015 to 2025 Across Clinical Applications, Quantitative Imaging, and Artificial Intelligence
Source: J Imaging. 2026 Aug 8;12(8):366. doi: 10.3390/jimaging12080366 (PMC13514230; doi:10.3390/jimaging12080366)
Supplement: Supplementary file 1 [file jimaging-12-00366-s001.zip › jimaging-4424965-supplementary.pdf]

Supplementary Methods

Detailed Search Strategies

The Scopus search was performed in the TITLE-ABS-KEY field on 31 May 2026 using the following query:

TITLE-ABS-KEY ("whole-body MRI" OR "whole body MRI" OR "whole-body magnetic resonance imaging" OR "whole body magnetic resonance imaging" OR "WB-MRI" OR "WBMRI" OR "whole-body diffusion-weighted imaging" OR "whole body diffusion weighted imaging" OR "whole-body DWI" OR "whole body DWI" OR DWIBS) AND PUBYEAR > 2014 AND PUBYEAR < 2026 AND (LIMIT-TO (DOCTYPE , "ar") OR LIMIT-TO (DOCTYPE , "re")).
Filters were applied to include only articles and reviews. The export contained 2,628 records.

A Topic search (TS) in Web of Science (WoS) on 31 May 2026 used the query:

TS = ("whole-body MRI" OR "whole body MRI" OR "whole-body magnetic resonance imaging" OR "whole body magnetic resonance imaging" OR "WB-MRI" OR "WBMRI" OR "whole-body diffusion-weighted imaging" OR "whole body diffusion weighted imaging" OR "whole-body DWI" OR "whole body DWI" OR DWIBS).

Search limits restricted results to the publication date range 2015-2025 and document types “Article” or “Review Article”. A total of 2,110 records were retrieved.

The following PubMed query was executed on 31 May 2026:

("whole-body MRI"[tiab] OR "whole body MRI"[tiab] OR "whole-body magnetic resonance imaging"[tiab] OR "whole body magnetic resonance imaging"[tiab] OR "WB-MRI"[tiab] OR "WBMRI"[tiab] OR "whole-body diffusion-weighted imaging"[tiab] OR "whole body diffusion weighted imaging"[tiab] OR "whole-body DWI"[tiab] OR "whole body DWI"[tiab] OR DWIBS[tiab]) AND ("2015/01/01"[PDAT] : "2025/12/31"[PDAT]).

The export yielded 1,455 records. Records were filtered to articles and reviews.

Record Screening and Eligibility

Records exported from each database were imported into a unified dataset. Automatic filters were re-applied after merging to retain articles and reviews published from 2015 through 2025 and to exclude conference abstracts, letters, editorials, corrections, and errata when document-type metadata were available. Reapplication of the normalized date and document-type filters removed 53 records outside 2015-2025 and 322 ineligible document types. After hierarchical deduplication, 3,139 unique metadata-eligible records underwent title and abstract relevance screening. Abstracts were available for 3,074 records; 65 were assessed using title and

INTERNAL

bibliographic metadata only. Records were retained when the title or abstract explicitly linked whole-body imaging to MRI, MR, diffusion-weighted MRI, PET/MRI, or another eligible whole-body MRI application and described substantive use, evaluation, analysis, or review of the method. Screening excluded 1,628 records: 1,556 without substantive WB-MRI use or evaluation, 60 ineligible publication types, and 12 WB-MRI acronym collisions. The final analytic corpus contained 1,511 publications.

Deduplication Process

Deduplication employed a hierarchical approach consistent with the main Methods. DOI was used first when available, followed by PMID, followed by normalized title-year-first author matching. Remaining near-duplicates were reviewed using first author, journal, year, and title similarity. If two records differed only in citation counts or minor metadata but represented the same article, they were merged. Duplicates were flagged, and the record with the most complete metadata, typically the Scopus entry, was retained. After deduplication and metadata eligibility filtering, 3,139 unique records remained for title and abstract relevance screening; 1,511 were retained in the final analytic corpus.

Data Extraction and Standardization

Fields extracted included title, year, journal, author list, first author, DOI, PMID, document type, citation counts, keywords, index keywords, affiliation strings and originating database. Titles were normalized by removing diacritics, converting to lower case and collapsing whitespace. Author lists were split by semicolons; initials were standardized (e.g., “Bamberg, Friedrich” to “Bamberg F.”). Citation counts were preferentially taken from Scopus when available. For records not indexed in Scopus, available citation metadata from Web of Science were used descriptively. Citation counts were treated as descriptive bibliometric indicators rather than standardized cross-database citation measures. For time-normalized analyses, citations per publication-year were calculated by dividing each record’s citation count by years since publication, using a minimum one-year denominator; the number of records with citation data was reported. Country information was derived from the last segment of each affiliation string. Exploratory institution strings were parsed from the first affiliation using the terms “University”, “Hospital”, “Institute”, “Centre”, and “Center”. Because ROR/GRID identifiers were unavailable and name variants could not be resolved reliably, institution rankings were not reported.

Keyword Processing and Co-occurrence Analysis

Author-supplied keywords and database index terms were combined. Keywords were converted to lower case; common stop words and demographic descriptors (e.g., “male”, “female”) were removed in substantive analyses. Synonyms were merged using a manually curated thesaurus: for example, “whole body MRI” and “WB-MRI” were treated as equivalent. Keyword co-occurrence node and edge tables were generated using Python/NetworkX for keywords appearing in at least ten articles. Weak co-occurrence edges and isolated nodes were filtered in the retained network files. For journal-scale legibility, Figure 3 presents the 30 most frequent

substantive keywords as a ranked two-panel display with exploratory rule-based colors. VOSviewer-compatible node and edge tables were exported for reproducibility.

Theme Tagging and Subgroup Definitions

Theme tagging was performed using prespecified keyword rules applied to titles, author keywords, and index keywords in the screened analytic dataset. Articles could receive multiple tags, so annual theme counts were overlapping rather than mutually exclusive. The rules were retained in the analysis code. No independent manual-validation sample or inter-rater reliability assessment was performed; the tags should be interpreted as exploratory, reproducible rule-based classifications rather than adjudicated categories. Exploratory subgroup analyses compared annual counts and both raw and time-normalized citation rates across themes.

Analysis Software

Data processing was performed in Python 3.11 using pandas for data manipulation and matplotlib for plotting. The PRISMA 2020 flow diagram was generated in R with the PRISMA2020 package. Citation and keyword analyses were supplemented by the R package Bibliometrix version 4.2. VOSviewer-compatible files were exported for reproducibility. Analysis scripts and derived aggregate outputs are available from the corresponding author upon reasonable request, subject to database licensing restrictions.

INTERNAL
